# Spontaneous Rupture of Uterine Vein in Twin Pregnancy

**DOI:** 10.1155/2013/596707

**Published:** 2013-12-19

**Authors:** Emek Doger, Yigit Cakiroglu, Sule Yildirim Kopuk, Bertan Akar, Eray Caliskan, Gulseren Yucesoy

**Affiliations:** Department of Obstetrics and Gynecology, Kocaeli University School of Medicine, Kocaeli, Turkey

## Abstract

*Objective*. Aim of our study is to present a case of a twin pregnancy following invitro fertilization cycle complicated with hemoperitoneum at third trimester. *Case*. A 26-year-old nulliparous pregnant woman at 32 weeks of gestation with twin pregnancy following invitro fertilization cycle complained of abdominal pain. After 48 hours of admission, laparotomy was performed with indications of aggravated abdominal pain and decreased hemoglobin levels. Utero-ovarian vein branch rupture was detected on the right posterior side of uterus and bleeding was stopped by suturing the vein. Etiopathogenesis of the present case still remains unclear. *Conclusion*. Spontaneous rupture of the uterine vessels during pregnancy is a rare complication and may lead to maternal and fetal morbidity and mortality. Diagnosis and treatment are based on the clinical symptoms of acute abdominal pain and laboratory tests of hypovolemic shock signs.

## 1. Introduction

Hemoperitoneum in pregnancy is a rare but a dramaticlife threatening complication that results from ruptured uterine vessels [1]. Even though the etiology is poorly understood, sudden rise in venous pressure turning out to vessel rupture and decidualised endometriosis invading vessel wall may be counted as responsible risk factors. In the English literature, approximately 150 cases of rupture of uterine vessels have been reported until now. Brosens et al. stated in their article that bleeding vessels were venous, arterial, or unknown (80%, 16% and 4%, resp.) [2]. Here we present a case of a twin pregnancy complicated with hemoperitoneum following in vitro fertilization cycle (IVF) as a result of utero-ovarian vein branch rupture.

## 2. Case

A 26-year-old nulliparous woman with a dichorionic-diamniotic twin pregnancy achieved by assisted reproductive techniques was admitted at 32 weeks of gestation. At admission, her complaints were epigastric pain and flank pain. Until that time, the patient had an uneventful pregnancy without history of vaginal bleeding, abdominal trauma, uterine contraction, or recent sexual intercourse. Her past medical history included neither abdominal surgery nor myoma uteri. On physical examination, the temperature was 36°C, the blood pressure was 110/70 mm Hg, the pulse was 92 beats per minute, and respiratory rate was 24 beats per minute and vital parameters were normal. At initial abdominal examination, she had no rebound tenderness. Pelvic examination was one cm dilated with a soft cervix. Cardiotocography showed irregular contractions with reactive fetal heart rate tracing. Hemoglobin (Hb) was 10.3 g/dL, hemotocrit level (Hct) was 31.4%, white blood cell count (WBC), C-reactive protein (CRP) level and other laboratory results were normal. Urinalysis revealed 100 mg/mL protein and leucocyte (++). Obstetrical ultrasonography examination revealed two otherwise healthy fetuses with apppropriate biometrical calculations and with normal amniotic fluid, Doppler flow velocimetry indices. Maternal abdominal ultrasound examination revealed minimal anechoic free fluid in the right lower quadrant but appendix was not observed. Transvaginal ultrasound examination revealed a 26 mm cervical length.

**Figure 1:**
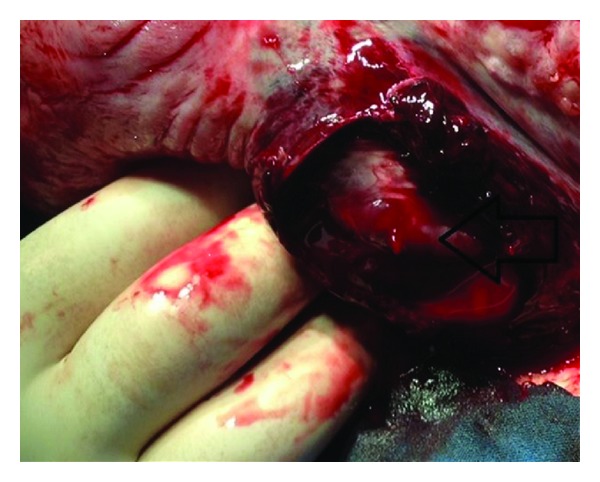
Arrow indicates actively bleeding vein.

**Figure 2:**
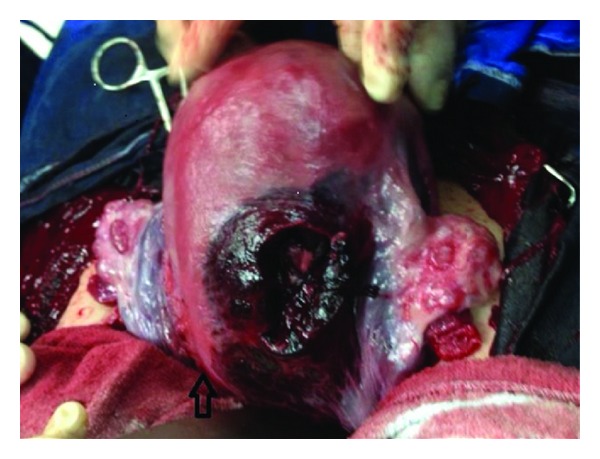
Artery was sutured with Vicryl 1.0. Arrow indicates incision side of the uterus.

After hospitalisation, steroid therapy betamethasone (Celestone Chronodose, 1 ml, Schering Plough, Germany) was performed for lung maturation. Nifedipine 10 mg 6 times a day (Nidilat, Sanofi Aventis, France) and then beta-agonists was administered for 24 hours for accelerated uterine contractions. Within 24 hours of admission the patient gradually complained of progressive nausea and vomiting. The patient was consulted to general surgery department, and oral intake was stopped, a nasogastric catheter was placed, a proton pump inhibitor was started intravenously, and an antibiotic regimen was administered (ceftriaxone 2 gr/day (Rocephine, Saba, Turkey)) and metronidazole 1 gr/day (Flagyl, Ecz.Baxter, Turkey) for suspicion of appendicitis. Whole abdominal and pelvic magnetic resonance (MR) imagings were performed and revealed a minimal intra-abdominal free fluid without any pathological signs. On the second day, abdominal pain with nausea and vomiting gradually increased but no rebound tenderness was experienced. Hg level decreased to 9.53 g/dL, htc level decreased to 29.4%, and WBC increased in value of 19.4 × 10^3^/UL. Since leucocyte counts increased gradually and symptoms became more obscure, surgery had been decided with the diagnosis of acute abdomen. Under spinal anesthesia, laparotomy was extended through pfannenstiel incision and 300–400 mL hemoperitoneum was observed in the peritoneal cavity. Pelvic exploration revealed blurish color on the posterior right side of the uterus, active bleeding from right uterine vessel, and a torn uterine serosa (Figure 1). A 8-9 apgar, 1760 gr, female fetus and a 8-9 apgar, 1730 gr, male fetus were born by a Cesarean section. The placenta was normal in appearance without retroplacental hematoma, ablation, or previa. Uterine vein branch was sutured with polyglactin 910 number 1.0. (Ethicon,Johnson & Johnson) (Figure 2). Postoperative period was uneventful without any necessity of blood transfusion, and the patient was discharged home on the second postoperative day.

## 3. Discussion

In 1950, the largest published series, a review of 75 cases due to utero-ovarian vessel rupture, reported a 49% of maternal mortality; however mortality rate declined to 3.6% by subsequent developments in medicine [1, 3]. Even though hemoperitoneum caused by utero-ovarian vessels rupture may occur in all trimesters, it mostly occurs in the third trimester; 61% occurred before labour, 18% was intrapartum, and 21% was in the puerperal period [3, 4]. Suggested etiologic factors are dilated utero-ovarian veins which have tortuous nature, lack of valves, increasing physiological demands of pregnancy, and muscular activities such as coughing, defecation, coitus, or pushing phase of second-stage labor that may cause sudden rise in venous pressure being predisposed to spontaneous rupture [5].

Sudden onset of abdominal pain, hypovolemic shock signs, and decreased hemoglobin levels indicate utero-ovarian vessels rupture such as other intra-abdominal bleeding causes. Amount, rapidity of blood loss and location of bleeding changes symptoms of hemoperitoneum [6]. Therefore the diagnosis of hemoperitoneum is so difficult before surgery. O'Connel and Pendiville reported a case with Cesarean section prompted by fetal distress with the lack of hemodynamic instability despite 1.51 liter hemoperitoneum [7]. Brosens et al. reviewed 25 cases of spontaneous hemoperitoneum in 12 publications and noted that 72% of spontaneous hemoperitoneum cases were nulliparous, with a mean age of 30, and also in all cases abdominal ultrasound examination failed to diagnose intraperitoneal bleeding [2]. Differential diagnosis cited uterine rupture, placental abruption, bleeding ectopic decidua, HELLP syndrome, splenic and hepatic artery aneurysm rupture, and perforated appendicitis.

In the English literature, until now there has been six reported twin cases of hemoperitoneum with stage IV endometriosis and one case with stage I endometriosis [8–13]. Chronic inflammation due to endometriosis which is influenced by progesterone is reported [14]. Decidualized endometriosis results in more fragile, vulnerable utero-ovarian vessels.

We present the fifth twin pregnancy following IVF treatment. Twin pregnancy incidence is increasing which may be due to frequent use of assisted reproductive technologies and has higher rates of obstetrical complications compared with single pregnancies. Therefore similar complications may increase. For the present case, the mechanism of rupture was indefinable, because of lack of endometriosis, adhesions, prior surgery, and myoma uteri. We do not know whether or not during ovum pickup procedure, damaged vessel wall or uterus was prone to rupture as a consequence of hemodynamic alterations of pregnancy such as increased cardiac output. However 3rd trimester ruptured vessel due to ovum pickup as a long term complication was not reported in the literature.

The preoperative diagnosis was so difficult because the patient had normal hemodynamic parameters and had a variety of clinical symptoms. Hemoglobin levels did not decrease rapidly which may be due to uterine pressure on the uterine vessel. When acute abdomen is diagnosed, hemoperitoneum should be considered in the differential diagnosis during gestation [15]. Nonspecific symptoms such as acute abdominal pain, vomiting, and maternal acute anemia are signals of hemoperitenoum that should be kept in mind. Close monitoring, instant diagnosis, and intervention should be done.

In conclusion, spontaneous uterine vessel rupture is a rare dramatic event in pregnancy. In a case presenting with severe abdominal pain with either fetal distress or maternal hemodynamic instability, spontaneous uterine venous rupture should be considered. Close observation and early intervention were essential for maternal and fetal survival.
